# Editorial: Understanding the Complex Phenomenon of Suicide: From Research to Clinical Practice

**DOI:** 10.3389/fpsyt.2018.00061

**Published:** 2018-03-01

**Authors:** Domenico De Berardis, Giovanni Martinotti, Massimo Di Giannantonio

**Affiliations:** ^1^NHS, Psychiatric Service of Diagnosis and Treatment, Department of Mental Health, Hospital “G. Mazzini”, ASL 4, Teramo, Italy; ^2^Department of Neurosciences and Imaging, Università degli Studi “G. d’Annunzio” Chieti, Chieti, Italy; ^3^Department of Pharmacy, Pharmachology and Clinical Sciences, University of Hertfordshire, Hatfield, United Kingdom

**Keywords:** suicide, knowledge, prevention, psychiatric disorders, crisis

**Editorial on the Research Topic**

**Understanding the Complex Phenomenon of Suicide: From Research to Clinical Practice**

Suicide is undoubtedly a worldwide major challenge for the public health. It is estimated that more than 150,000 persons in Europe die as a result of suicide every year and in several European countries suicide represents the principal cause of death among young people aged 14–25 years (1). Moreover, it has been reported that the standardized death rate for intentional self-harm in the European Union is higher in the elderly (≥65 years) and over than for younger people (2, 3). The highest suicide rates in the world (21–35/100,000) have been found in the countries of Eastern Europe where the numbers of deaths due to suicide may be considered as an emergency (4, 5). Moreover, in a recent study, Olfson et al. (6) have reported that also suicide attempts in USA increased significantly from 0.62 to 0.79% among the adult population aged 21 years and older, based on representative community samples recruited from 2004 to 2005 and 2012 to 2013.

It is true that suicide is a complex (and yet not fully understood) phenomenon and may be determined by the interaction between various factors, such as neurobiology, personal and familiar history, stressful events, sociocultural environment, etc. (7). In fact, suicide results from many multifaceted social and cultural factors and is more likely to happen during periods of socioeconomic, family, and individual crisis situations (e.g., death of a loved one, loss of employment, etc.) (8). However, suicide remains an important problem that violently hits the world public health and suicide prevention should be always a priority even in those nations where suicide rates are lower. In fact, it is necessary to underline that several strategies aimed to reduce suicide when implemented are often effective and, for governments and health economics, quite inexpensive when compared to the direct and indirect costs of lives’ loss (9, 10).

The suicide is always a plague for the population at risk and one of the most disgraceful events for a human being. Moreover, it implies a lot of pain often shared by the relatives and persons who are close to suicide subjects (11, 12). Furthermore, it has been widely demonstrated that the loss of a subject due to suicide may be one of the most distressing events that may occur in mental health professionals resulting in several negative consequences, such as burnout, development of psychiatric symptoms (anxiety and depression), and lower quality of life and work productivity (13–15). In addition, the loss of a patient by suicide may have for the physicians and mental health professionals’ medico-legal consequences that may increase the burden (16, 17).

It is important to underline that the majority of suicides typically occurs in the context of a psychiatric disorder and/or as a consequence of severe medical diseases (18–20). Thus, all people who work in the context of public and mental health must know the most important literature about suicide and be informed of risk factors and warning signs of it (21). Mental health is supposed to be at a greater risk of suicide, as individuals with mental disorders (mostly mood disorders) are an extremely vulnerable population (22–24). It is important to remark that many subjects with (but also without) psychiatric disorders often try to seek help before attempting or completing suicide (25, 26). This “crying for help” may be often covert and psychiatrist must be trained to early recognize the warning signs of an imminent suicide (27). It is remarkable that the topic of suicide is often forgotten or neglected in psychiatric residency and training and merely limited to consider it a simple epiphenomenon of a psychiatric disorder (28). We firmly believe that the introduction of “Suicidology” as a teaching subject in all universities and psychiatry schools should be warranted in order to provide an adequate training on this topic to all who will deal with medical and psychiatric illnesses.

Moreover, the global distressing actual conditions due to economic crisis may contribute further to increase suicidal ideation and/or behaviors. In 1897, the French suicidologist Emile Durkheim, in his well-known book “Le Suicide. Étude de Sociologie,” examined the relationship between industrial and financial crises and suicide rate. Several studies have recently evaluated the relationships between suicide and economic crisis but this association is often unclear and may be mediated by the development of psychiatric disorders or other factors (29–31).

From these data, it is clear that suicide prevention is a worldwide priority and every effort should be made in order to improve early recognition of imminent suicide, manage suicidal subjects, and strengthen suicide prevention strategies. In our opinion, the first step of prevention is the improvement of knowledge in the field: this was the aim of this present special issue on Frontiers in Psychiatry. In the special issue, several papers have contributed to the suicide knowledge from several viewpoints.

From an historical perspective López-Muñoz and Cuerda-Galindo reviewed suicide in inmates in Nazis and Soviet concentration camps and showed that the incidence of suicide in Nazi camps could be up to 30 times higher than the general population and was also much higher than in Soviet special camps even if data interpretation is very controversial.

From a psychopathological point of view, several contributions were remarkable. Hilario Blasco-Fontecilla et al. suggested that both non-suicidal self-injury and suicidal behavior can be conceptualized as addictions and if some individual’s self-harming behaviors are so conceptualized, treatment approaches could be taken this into account and tailored to this addiction. Verrocchio et al. conducted a systematic review analyzing the relationship between mental pain and suicide and found that mental pain was a core clinical factor for understanding suicide, both in the context of mood disorders and independently from depression. Falgares et al. evaluated a sample of 340 high-school students to evaluate whether self-criticism and dependency mediate the relationship between insecure attachment styles and suicidality. They found that both self-criticism and dependency were significant mediators in the relationship between attachment anxiety and suicidality, whereas only self-criticism mediated the relationship between attachment avoidance and suicidality. De Berardis et al. reviewed the role of alexithymia in triggering suicidal ideation and behavior in psychiatric patients. They underlined the importance of alexithymia screening in everyday clinical practice, as well as the evaluation of clinical correlates of alexithymic traits that should be integral parts of all disease management programs and, especially, of suicide prevention plans and interventions.

From a neural perspective, Deshpande et al. tried to understand the neural substrates underlying the gender differences in the rate of fatal suicidal behavior keeping in mind the Interpersonal–Psychological Theory of Suicide. They conclude that suicidal desire generally leads to fatal/decisive action in males, while, in females, it manifests as depression, ideation, and generally non-fatal actions and this has different neural basis. The importance of these finding was remarked by Professor Gonda’s commentary on the Deshpande’s paper.

From a social psychiatry perspective, the paper of Carpiniello and Pinna focused on the complex relationships that may exist between suicidal behavior and stigmatizing attitudes. They pointed out a reciprocal relationship between stigma and suicide: suicide may cause stigmatizing attitudes, but stigma toward mental disorders may be a risk factor for suicidality. Therefore, we must fight a battle against stigma to sustain self-esteem, reduce isolation, and empower those suffering from perceived or internalized stigma.

From an epidemiological point of view, two papers were of particular interest. Ventriglio et al. reviewed the available data on relationships between that first episode of psychosis (FEP) and the suicide risk. They concluded that patient with FEB may be at risk of suicide and the most relevant risk factors for suicide in FEP were associated with age of onset of psychotic symptoms, duration of untreated psychosis, some demographic characteristics, psychopathology, trauma, and insight. Orsolini et al. aimed at providing a focused review about epidemiological data, risk and protective factors, and an overview about the main clinical correlates associated with the suicidal behavior during the pregnancy and postpartum period. Despite a common belief that pregnancy and postpartum period may be at lower risk of suicide, they found that suicide still remains one of the most common leading causes of maternal death during the 1 year following delivery. The screening during the perinatal period (particularly during pregnancy) represents an essential clinical tool for identifying women at higher risk of perinatal suicidality.

From a clinical perspective, Morales et al. tried to gain a deeper understanding of the state of suicide risk by determining the combination of variables that distinguishes between groups with and without suicide risk, evaluating 707 patients consulting for mental health issues in three health centers in Greater Santiago, Chile. They individuated the interactions among a group of variables associated with suicidal ideation and behavior and, using these variables, it was possible to generate four decision trees that can distinguish between groups with and without suicidal behavior. Gramaglia et al. aimed to compare the severity of depressive symptoms in depressed inpatients admitted after an attempted suicide and those admitted for any other reason and to assess the severity of suicide attempts and the management of suicidal risk in clinical settings. They found no correlations among psychiatric diagnosis, psychiatric and physical comorbidity, severity of depressive symptoms, and suicidal behavior. On the other hand, antidepressant therapy was found to protect against suicide attempts. Schneider et al. used the Traumatic Brain Injury (TBI)-4, a four-question screener used to identify those with a probable lifetime history of TBI, administered to 1,097 Veterans at the time of mental health intake, to determine whether a positive screen on the TBI-4 was associated with increased risk for suicide attempt within 1-year post screening. They found that Veterans with a positive TBI screen at mental health intake had a higher proportion of suicide behavior reports in Veteran electronic medical records than those who screened negative for TBI.

From a psychosomatic perspective Conti et al. carried out a systematic review analyzing the relationship between Diabetes Mellitus (DM) and suicide by providing a qualitative data synthesis of the studies. DM was found to be significantly associated with a marked increase in suicidal behaviors and suicidal ideation, especially in patients with depressive symptoms. Authors remark that health-care professionals need to be aware of the higher suicidal risk in some patient subgroups based on the clinical characteristics of DM.

Concerning the “hot” topic of suicide prevention, Klimes-Dougan et al. evaluated how the suicide prevention public service announcements may impact help-seeking attitudes. They conclude that “The Message Makes a Difference.” In fact, the results of the study provided some optimism that carefully crafted billboard messages may favorably influence help-seeking attitudes of participants.

In conclusion, in this special issue, we tried to improve the knowledge on suicide from several different perspectives. The number of published papers demonstrated a great worldwide attention to the topic. Today, the suicide is a major concern and we firmly believe that improving knowledge may also improve prevention strategies. This is our hope, and every subject at risk of suicide deserves all efforts that we can put in place, as clinicians and as human beings.

## Author Contributions

We state that (1) all authors have read the paper and approved the data and the conclusions presented therein; (2) each author believes that the paper represents honest work; (3) all authors have contributed to the present paper with equal effort; and (4) no financial support was given for this editorial.

## Conflict of Interest Statement

The authors declare that the research was conducted in the absence of any commercial or financial relationships that could be construed as a potential conflict of interest.
